# Analysis of Polymorphisms rs7093069-IL-2RA, rs7138803-FAIM2, and rs1748033-PADI4 in the Group of Adolescents With Autoimmune Thyroid Diseases

**DOI:** 10.3389/fendo.2020.544658

**Published:** 2020-10-23

**Authors:** Beata Sawicka, Hanna Borysewicz-Sańczyk, Natalia Wawrusiewicz-Kurylonek, Tommaso Aversa, Domenico Corica, Joanna Gościk, Adam Krętowski, Małgorzata Waśniewska, Artur Bossowski

**Affiliations:** ^1^ Department of Pediatrics, Endocrinology, Diabetology, with Cardiology Division, Medical University of Bialystok, Białystok, Poland; ^2^ Department of Endocrinology, Diabetes with Internal Medicine, Medical University of Bialystok, Białystok, Poland; ^3^ Department of Human Pathology of Adulthood and Childhood, University of Messina, Messina, Italy; ^4^ Software Department, Faculty of Computer Science, Białystok University of Technology, Bialystok, Poland

**Keywords:** Hashimoto’s thyroiditis, Graves’ disease, gene polymorphism, FAIM2, PADI4, Il-2RA

## Abstract

**Introduction:**

The pathogenesis of autoimmune thyroid diseases is complicated and not completely known. Among the causes of thyroid autoimmunity, we distinguish genetic predisposition and environmental factors. Graves’ disease and Hashimoto’s thyroiditis are associated with a disturbance of immune tolerance of thyroid antigen molecules. The IL2RA gene is located on chromosome 10 and encodes the interleukin 2 receptor (IL2RA), which is expressed by the regulatory T-cells (Tregs) responsible for suppression. It has been shown that this gene and its polymorphism occur in people with various autoimmune diseases (*e.g.* type 1 diabetes mellitus, rheumatoid arthritis, Graves’ disease, or multiple sclerosis). The FAIM2 gene is located on chromosome 12 and encodes the molecule involved in the apoptosis inhibition process. The PADI4 gene is located on chromosome 1, and its expression is associated with activation of T-cells, differentiation of macrophages, which leads to increased inflammation.

**Aim:**

The aim of the study was to analyze the polymorphisms of the IL-2RA (rs7093069), FAIM2 (rs7138803) and PADI4 (rs1748033) genes and their correlation to thyroid hormones and anti-thyroid antibodies in pediatric patients with Graves’ disease and Hashimoto’s thyroiditis compared to the control group.

**Material and Methods:**

The study was performed in 180 patients with GD (mean age 16.5 ± 2), 80 with HT (mean age, 15.2 ± 2.2), and 114 children without any autoimmune diseases (mean age 16.3 ± 3) recruited from the endocrinology outpatient clinic. Three single nucleotide polymorphisms (SNPs): rs7138803-FAIM2, rs7093069-IL-2RA, and rs1748033 PADI4 were determined by TaqMan SNP QuanStudio 12K Flex-OpenArray genotyping with PCR and correlated to thyroid hormones and anti-thyroid antibodies.

**Results:**

Rs7090369-IL-2RA allele T was more frequent in patients with AITDs (33.7% in GD *vs* 28.7% in HT, p = 0.077, OR = 1.52) compared with healthy children (25%). Allele T of that gene predisposes to the occurrence of autoimmune thyroid diseases, especially GD and TT genotype gives a statistically significant 5.2 times higher risk of GD (p = 0.03, OR = 5.26) and increased risk of HT (p = 0.109, OR = 4.46). Allele A rs7138803-FAIM2 is more frequent in patients with GD (p = 0.071, OR = 1.45) and HT (p = 0.028, OR = 1.8). In our data the presence of GG genotype of that gene significantly reduces the risk of autoimmune thyroid diseases (p = 0.05, OR = 0.42). Allele C rs1748033PADI4 and its CC genotype were more frequent in patients with autoimmune thyroid diseases, but it was not statistically significant. The occurrence of CT genotype significantly reduces the risk of HT (p = 0.03, OR = 0.4).

**Conclusions:**

1). Polymorphisms rs7138803-FAIM2 and rs1748033-PADI4 are more frequent in patients with autoimmune thyroid diseases, more frequent in patients with Hashimoto’ thyroiditis, but the occurrence of GG rs7138803-FAIM2 genotype could reduce the risk of thyrocyte apoptosis inhibition. 2). The TT rs7093069-IL2RA genotype may increase the risk of autoimmune thyroid diseases. 3). Analysis of polymorphisms of given genes in clinical practice will allow to determine predisposition to autoimmune thyroid disease development, to find symptoms of thyroid gland dysfunction earlier and to use appropriate treatment.

## Introduction

Nowadays we observe an increase of newly diagnosed autoimmune thyroid diseases (AITD), like Graves’ disease (GD) and Hashimoto’s thyroiditis (HT) in different age groups. The pathogenesis of thyroid diseases is still unclear. Significant progress has been made in our understanding of the genetic and environmental triggers contributing to AITD (1). A number of molecular changes occur in genetically predisposed individuals in order to develop clinical symptoms (2, 3).

GD and HT are associated with a disorder of immune tolerance of thyroid antigen molecules. Both are involved with reduced regulatory T-cell (Treg) function. We distinguish a number of different genes encoding proteins included in the structure of the thyroid cell, elements of the immune system and those responsible for modulation of the apoptosis process. Among genetic factors we could divide: genes which coding thyrocyte elements (Tg, TSHR, SEPS1), genes encoding immunomodulators of thyrocyte antigen molecules responsible for the peripheral response (IL-2RA, FOXP3, FCRL3) (4) and responsible for T lymphocyte activation and antigen presentation (CD40, PTPN22, CTLA-4, HLA-B8, DR-3, DR-4, DR-5, PADI) (5, 6), and genes responsible for activating/inhibiting apoptosis (FAIM2).

Gene Il-2RA (interleukin 2 alpha-receptor gene) is located on the short arm of chromosome 10 (p15.1) and coded protein also called CD25, which forms an alpha-receptor chain for interleukin 2 (high-affinity alpha subunit—CD25—of the interleukin-receptor). The molecular weight of CD25 is approximately 55 kDa. This protein plays an essential role in the T lymphocyte response to IL-2, which is the main growth factor for these cells—CD25 expression is important for proliferation, longer life expectancy and T-cell function. CD25 occurs on the surface of maturing T and B lymphocytes; it undergoes transient expression on activated T and B lymphocytes; it constitutively occurs on regulatory T lymphocytes (Tregs), which inhibit activation of autoreactive T lymphocytes. It was shown that the polymorphism of this gene occurs in people with various autoimmune diseases (*e.g.* diabetes mellitus type 1, rheumatoid arthritis, Graves’ disease, sclerosis multiplex) (7–13).

Gene FAIM2 (Fas apoptotic inhibitory molecule 2 gene) is located on the long arm of chromosome 12 (q13.12). The molecular weight of FAIM2 is approximately 35 kDa. This gene codes the molecule involved in the apoptosis inhibition process (inhibits Fas-mediated). It regulates apoptosis in neurons by interfering with the activation of caspase-8. It can play a role in cerebellum development. The polymorphism of the molecular coding gene is associated with giant obesity and diabetes mellitus type 2 and increases the risk of cardiovascular diseases (14–19).

Gene PADI4 (Peptydil Arginine Deiminase 4) is located on the short arm of chromosome 1 (p36.13). The molecular weight of PADI4 is approximately 74 kDa. This gene codes the enzymatic proteins responsible for the deamination of arginins. It plays a role in the development of granulocytes and macrophages, leading to inflammation and immune response. It catalyzes the citrilution/deimination of arginine residues of proteins such as histones, thus playing a key role in the histone code and regulation of stem cell maintenance. It is found in the synovial membrane of people with rheumatoid arthritis (20).

The aim of the study was to analyze the polymorphisms of the IL-2RA (rs7093069), FAIM2 (rs7138803) and PADI4 (rs1748033) genes and their correlation to thyroid hormones and anti-thyroid antibodies in pediatric patients with Graves’ disease and Hashimoto’s thyroiditis compared to the control group.

## Materials and Methods

The study was performed in 180 patients with GD (mean age 16.5 ± 2), 80 with HT (mean age, 15.2 ± 2.2), and 114 children without any autoimmune diseases (mean age 16.3 ± 3) recruited from two pediatric endocrinology outpatient clinics. The diagnosis of autoimmune thyroid diseases was based on medical history, physical examination, laboratory, and ultrasound investigations (21, 22). Clinical diagnosis of hyperthyroidism in GD is confirmed by elevated thyroid hormones in serum and suppression of TSH to values close to zero with positive antibodies against receptor for thyroid-stimulating hormone (TRAb = anti-TSH), positive anti-thyroid peroxidase antibodies (aTPO) and anti-thyroglobulin antibodies (aTG) (23, 24). In clinically evident hypothyroidism in HT, serum TSH levels are elevated at reduced concentrations of thyroid hormones (fT4, fT3), usually accompanied by elevated thyroid antibodies (aTPO and/or aTG, rarely blocking anti-TSH). In patients with concomitant nodular goiter, fine-needle aspiration biopsy (FNAB) was also performed, and we excluded thyroid carcinomas. All patients had appropriate therapy for autoimmune thyroid pathology. Patients with GD were treated with methimazole and b-blockers orally. In patients with HT in therapy was used l-thyroxine orally. We excluded other endocrinopathies, extra-thyroid autoimmune diseases or non-endocrine autoimmune diseases. The control group consisted of 114 healthy children with no personal or family history of any AITDs. They were euthyroid and negative for thyroid antibodies. All controls had normal thyroid gland in ultrasonography. Before enrollment, all patients and controls and all children over 16 years old signed informed consents. The protocol for the study was approved by the Local Bioethical Committee at the Medical University of Bialystok.

### Assessment of the Thyroid Hormone Concentration and Anti-Thyroid Antibody Titers

Blood for analysis was collected in the morning from the basilic vein. Serum levels of free thyroxine (fT4), free triiodothyronine (fT3), and TSH were determined on electrochemiluminescence ‘ECLIA’ with Cobas E411 analyzer (Roche Diagnostics). Normal values for fT4 ranged between 1.1 and 1.7 ng/dl, for fT3 between 2.3 and 5.0 pg/ml, and for TSH between 0.28 and 4.3 (µIU/L). TR-Ab, anti-TPO and anti-TG antibodies were measured in all samples using ECLIA with Modular Analytics E170 analyzer (Roche Diagnostics). The positive values for anti-thyroid antibodies were: >1.75 U/L for TR-Ab, >34 IU/ml for anti-TPO-Abs and >115 IU/ml for anti-TG-Abs.

### Genotyping

DNA was extracted from the leukocytes deriving from peripheral blood using classical salting-out method. It was performed according to the manufacturer’s protocol. The three analyzed SNPs rs 709369 in the IL-2RA gene, rs 7138803 in the FAIM2 gene, and rs1748033 in the PADI4 gene were determinated byTaqMan SNP QuanStudio 12K Flex-OpenArray genotyping with PCR. SNP analysis was performed twice, by the commonly used instructions. As a negative control, we used a molecular grade water.

### Statistical Analysis

To assess any relationship between allele or genotype occurrence and patient’s status median unbiased estimator (mid-p) of odds ratio (as well as its 95% exact confidence interval), the exact confidence interval (CI) and associated p value obtained with the use of Fisher’s exact test, both obtained with the use of the mid-p method were used (25). To determine statistically significant differences between groups defined by genotypes and quantitative features, either parametric or non-parametric methods were used depending on fulfilling the normality and homogeneity of variance assumptions. Due to the issue of multiple testing during the *post-hoc* analysis, false discovery rate p value adjustment method was applied (26). Measure D’ of linkage disequilibrium was used as proposed in (27). The p value of <0.05 was considered to be significant for all calculations. The R software environment was exploited for all calculations (28).

## Results

Age and anthropometric parameters in the study groups of children with GD, HT compared to the control group did not have statistically significant differences (**Table 1**).

**Table 1:** Clinical characteristics of patients with Graves’ disease (GD) and with Hashimoto’s thyroiditis (HT) and control group.

	GD (mean ± SD)	p*	HT (mean ± SD)	p**	Control group
Female/Male	180 (135/45)		80 (59/21)		114 (88/26)
Age (years)	16.5 ± 2	NS	15.2 ± 2.2	NS	16.3 ± 3
Weight (kg)	55.19 ± 2.39	NS	58 ± 5.28	NS	60.9 ± 7.8
Height (cm)	162.1 ± 2.6	NS	154.2 ± 4.1	NS	160 ± 8
BMI (kg/m^2^)	21.1 ± 2.1	p < 0.012	24.45 ± 1.33	NS	23.78 ± 2.5
fT4 (ng/dl)	3.6 ± 1.4	p < 0.001	1.21 ± 0.03	NS	1.1 ± 0.17
fT3 (pg/ml)	7.19 ± 1.65	p < 0.001	3.59 ± 0.69	NS	3.79 ± 0.18
TSH (mIU/L)	0.37 ± 0.1	p < 0.01	6.45 ± 3.27	p <0.02	3.04 ± 0.72
Anti-TSH (U/l)	11.56 ± 2.11	p < 0.001	0.5 ± 0.32***	NS	0.4 ± 0.2
aTG (IU/ml)	347.49 ± 86.7	p < 0.001	389.8 ± 245.34	p <0.001	41.6 ± 12.1
aTPO(IU/ml)	431.97 ± 58.12	p < 0.001	531.5 ± 460.93	p <0.001	26.72 ± 6.8
Treatment	methamizole/ b-blocker		l-thyroxine		none

NS, no statistical significance.

p*, statistical significance between patients with GD and controls.

p**, statistical significance between patients with HT and controls.

anti-TSH***, anti-TSH antibodies levels were analyzed in a selected group of HT patients. (n = 43).

### Results for IL-2RA (rs7093069)

Our study shows that rs7093069 T alleles were more frequent in patients with AITDs (33.7% in GD *vs* 28.7% in HT, p = 0.077, OR = 1.52) compared with healthy children (25%) (**Table 2**). Allele T predisposes to the occurrence of autoimmune thyroid diseases, especially GD, and T/T genotype gives a statistically significant 5.2 times higher risk of GD (p = 0.03, OR = 5.26) and increased risk of HT (p = 0.109, OR = 4.46). Comparing both groups of autoimmune diseases: patients with GD and HT allele T, it is more common in patients with GD (33.7 *vs* 28.7%, p = NS). There was no statistically significant correlation between rs7093069-IL-2RA thyroid hormones levels and anti-thyroid antibodies in the studied groups (data not supplied).

**Table 2:** Genotype and allele frequencies for IL-2RA gene polymorphism (rs7093069) in groups with Graves’ disease (GD) and with Hashimoto’s thyroiditis (HT) compared to control group.

Group	Patients with GB (n = 180)	p (95%CI)/OR	Patients with HT (n = 80)	p (95%CI)/OR	Control group (n = 114)
Allele C	175 (66.2%)	NS	67 (71.2%)	NS	114 (75%)
REF		p = 0.077;			
T	89 (33.7%)	OR = 1.52	27 (28.7%)	p = 0.05 OR = 1.2	38 (25%)
Genotype					
CC CT TT	51 (38.6%) 73 (55.3%) 8 (6.1%)	NS NS p = 0.03 OR = 5.26	23 (48.9%) 21 (44.6%) 3 (6.3%)	NS NS p = 0.109 OR = 4.46	38 (50%) 38 (50%) 0 (0%)

P, p-value; REF., reference. NS, p-value > 0.05. OR, odds ratio. 95% CI, 95% confidence interval for odds ratio.

### Results for FAIM2 (rs7138803)

In the study groups we reported that allele A is more frequent in patients with GD and HT. Allele A was found in 44.7% of patients with GD (p = 0.071, OR = 1.45), in 50% of patients with HT (p = 0.028, OR = 1.8), and in 35.6% of healthy children (**Table 3**). In our data the presence of GG genotype significantly reduces the risk of autoimmune thyroid diseases (p = 0.05, OR = 0.42). There were no significant differences in the frequency of A and G alleles and their genotypes when comparing the groups of patients with autoimmune diseases (data not included). A statistical correlation between rs 7138803 FAIM2 gene polymorphism and aTPO antibodies in patients with GD was found (p < 0.05). There was no statistically significant correlation between rs7138803 FAIM2, thyroid hormone levels, and other anti-thyroid antibodies in the studied groups (data not supplied).

**Table 3:** Genotype and allele frequencies for FAIM2 gene polymorphism (rs7138803) in groups with Graves’ disease (GD) and with Hashimoto’s thyroiditis (HT) compared to control group.

Group	Patients with GB (n = 180)	p (95%CI)/OR	Patients with HT (n = 80)	p (95%CI)/OR	Control group (n = 114)
Allele A	126 (44.7%)	p = 0.071	51 (50%)	p = 0.028	57 (35.6%)
REF		OR = 1.45		OR = 1.8	
G	156 (55.3%)	NS	51 (50%)	NS	103 (64.3%)
Genotype					
AA AG GG	45 (21.27%) 66 (46.8%) 45 (31.9%)	NS NS p = 0.05 OR = 0.42	12 (23.5%) 27 (53%) 12 (23.5%)	NS NS p = 0.02 OR = 0.28	9 (11.2%) 39 (48.7%) 32 (40%)

P, p-value; REF., reference. NS, p-value >0.05. OR, odds ratio. 95% CI, 95% confidence interval for odds ratio.

### Results for PADI4 (rs1748033)

Allele C rs 1748033 PADI4 and its CC genotype were more frequent in patients with autoimmune thyroid diseases, but it was not statistically significant. The occurrence of CT genotype significantly reduces the risk of HT (p = 0.03, OR = 0.4) (**Table 4**). Allele T and its CT genotype occur more frequently in this group of patients with GD, but it is not a significant correlation. The positive correlation of rs1748033 PADI4 with anti-TSH antibodies in patients with GD (p = 0.05) and positive correlation of this gene with aTPO antibodies in patients with HT were found (p = 0.001). It is worth noting that there were no statistically significant correlations between gene polymorphisms and thyroid hormone levels in patients with autoimmune thyroid diseases (data not supplied).

**Table 4:** Genotype and allele frequencies for PADI4 gene polymorphism (rs1748033) in groups with Graves’ disease (GD) and with Hashimoto’s thyroiditis (HT) compared to control group.

Group	Patients with GB (n = 180)	p (95%CI)/OR	Patients with HT (n = 80)	p (95%CI)/OR	Control group (n = 114)
Allele C	155 (74.8%)	NS	78 (76.5%)	NS	107 (69.5%)
REF					
T	69 (25.2%)	NS	24 (23.5%)	NS	47 (30.5%)
Genotype					
CC	78 (56.9%)	NS	34 (66.7%)	NS	39 (50.6%)
CT	49 (35.8%)	NS	10 (19.6%)	p=0.03	29 (37.7%)
				OR=0.4	
TT	10 (7.3%)	NS	7 (13.7%)	NS	9 (11.7%)

P, p-value. REF., reference. NS, p-value >0.05. OR, odds ratio. 95% CI, 95% confidence interval for odds ratio.

## Discussion

In recent years, many reports have been published confirming the various genes in the development of autoimmune thyroid diseases. There are still very few reports of children assessing chosen genes as a risk autoimmune endocrine disorder (2, 3, 5).

An important development of autoimmune diseases is the disturbed immune system. Physiologically correct investigation and removal of the “foreign” antigen ensure the safety of the owned tissues and organs. Autoreactive lymphocytes are cloned in thymus, while in peripheral organs of the immune system, tolerance is achieved by energy or active suppression with Tregs regulatory (suppressor) lymphocytes. IL-2-cytokine is the most important growth factor for T lymphocytes. Moreover, this cytokine has a positive effect on the immune response because, after stimulation of T lymphocyte, it induces the appearance of molecules on its surface that enable apoptosis of this cell. Many studies have confirmed the association of IL-2 level in serum with many autoimmune diseases, for example, diabetes mellitus type 1 (7), pediatric atopic dermatitis (8), alopecia areata (9), autoimmune thyroid diseases (10), or multiple sclerosis (11). There are only few studies evaluating alleles and individual genotypes of the IL-2 gene in particular diseases (12). It has been shown that the alleles T in rs11938795 Il and Gin rs6822844 Il 2 were significantly associated with a higher risk of colorectal carcinoma in adults (13). Our study has showed that rs7093069 T alleles were more frequent in patients with AITDs compared with healthy children. Genotype TT gave a statistically significant 5.2 times higher risk of GD and increased risk of HT.

As it turns out, there are various immunomodulators that strictly control cellular functions. For example, the citrullination is a process in which arginine is deminated to citrulline. It is catalyzed by a group of hydrolyses called arginine protein deiminases (PADs), from which, till now, five isoforms have been identified. Hypercitrullinating, as a result of increased expression or activity of PAD, is associated with autoimmune diseases such as rheumatoid arthritis, lupus, Alzheimer’s disease, ulcerative colitis, multiple sclerosis, and certain cancers (29). PADI4 is found in various cells, including granulocytes, lymphocytes, monocytes and macrophages (30). One of the theories of the autoimmune process is the production of antibodies against citrulline proteins. Peptidylarginine deiminase 4 (PADI4) catalyzes the citrullines of histones and thus regulates the maintenance of stem cells. This process results in extracellular neutrophil traps (NETs), which as citrullinated proteins, are the target of autoantibodies in the treatment of inflammation and arthritis (31). PADI4 was found in the differentiation of macrophages and its role in inflammatory reactions (32). So far, there are no studies evaluating the polymorphism of the gene encoding PADI4 in humans with autoimmune diseases of the thyroid gland. We found that patients with allele C rs1748033 PADI4 and its CC genotype predisposes to the occurrence of autoimmune thyroid diseases.

Apoptosis plays an important role in the pathomechanism of autoimmune thyroid diseases. It is one of the forms of programmed cell death. In Hashimoto’s thyroiditis, cytokines released by macrophages and Th1 lymphocytes induce mass regulation by the increase of expression of CD95 molecules on the surface of thyroid cells. This stimulation activates the programmed death of these cells when Fas ligand is present on their surface as a result of self-destruction mechanism. In Graves’ disease, Th2 lymphocytes are infiltrated and IL-4 and IL-10 are produced, causing the expression of anti-apoptotic molecules and also, their resistance to apoptosis through CD95. Thyroid cells, both Graves’ disease and Hashimoto’s disease, showed a strong expression of Fas ligand together with a receptor for Fas. As a result of combining the Fas receptor with its ligand, intracellular areas are initiated, which activate cascades responsible for cell death. There are many molecules that influence the apoptosis process. One of them is the FAIM2 molecule. Many studies suggest that the polymorphism of this gene is associated with obesity and the development of type 2 diabetes (14). A multicenter study of more than 13,000 children aged 2–18 with BMI above 95 percentile years confirmed the occurrence of this gene polymorphism in obese children (15). The variant of gene FAIM2-rs7138803 is associated with BMI z-scores in children over 6 years (16). On the other hand, the Mexican researchers have not confirmed that kind of association in obese children without metabolic disorders (17). It is known that the obesity gene, the apoptotic suppressing molecule Fas 2 (FAIM2), is regulated by nutritional status, and the promoter FAIM2 methylation levels are significantly related to overweight. An interesting study was conducted by Chinese researchers, who studied the influence of different lifestyles on methylation changes in obese and lean children. They have showed that lifestyle (17) might have an impact on FAIM2 (18), but it has been independently associated with dyslipidemia (19). The polymorphisms of this gene in people with thyroid diseases are till now unknown. In patients with HT, thyroid cells are destroyed as a result of cytotoxic action of T lymphocytes and increased process of apoptosis. Even though in our study we reported that allele A of gene FAIM2-rs7138803 is statistically significantly more frequent in patients with HT, the presence of GG genotype significantly could reduce the risk of autoimmune thyroid diseases. Because of the small group, this requires further research.

There are no studies of these genes’ polymorphisms in adolescents with autoimmune thyroid diseases in the literature. To sum up, analysis of polymorphisms of presented genes in clinical practice could allow for determining the predisposition to autoimmune thyroid disease development, to find symptoms of thyroid gland dysfunction earlier, and to use appropriate treatment, but it still requires a lot of research to improve the knowledge on this topic.

## Conclusions

1. Polymorphisms rs7138803-FAIM2 and rs1748033-PADI4 are more frequent in patients with autoimmune thyroid diseases, more frequent in patients with Hashimoto’ thyroiditis, but the occurrence of GG rs7138803-FAIM2 genotype could reduce the risk of thyrocyte apoptosis inhibition.
2. The TT rs7093069-IL2RA genotype may increase the risk of autoimmune thyroid diseases.
3. Analysis of polymorphisms of given genes in clinical practice will allow for determining predisposition to autoimmune thyroid disease development, for finding symptoms of thyroid gland dysfunction earlier, and for using appropriate treatment.

## Data Availability Statement

The raw data supporting the conclusions of his article will be made available by the authors, without undue reservation.

## Ethics Statement

The studies involving human participants were reviewed and approved by Bioethics Committee of Medical University in Bialystok. Written informed consent to participate in this study was provided by the participants’ legal guardian/next of kins.

## Author Contributions

The authors equally participated in the creation of the work. All authors contributed to the article and approved the submitted version.

## Conflict of Interest

The authors declare that the research was conducted in the absence of any commercial or financial relationships that could be construed as a potential conflict of interest.
